# Global hotspots and research trends of radiation-induced skin injury: a bibliometric analysis from 2004 to 2023

**DOI:** 10.3389/fonc.2024.1430802

**Published:** 2024-08-26

**Authors:** Yungang Hu, Lu Yu, Weili Du, Xiaohua Hu, Yuming Shen

**Affiliations:** Department of Burns and Plastic Surgery, Beijing Jishuitan Hospital, Capital Medical University, Beijing, China

**Keywords:** radiation-induced skin injury, radiotherapy, bibliometric analysis, Citespace, VOSviewer

## Abstract

**Background:**

Radiation therapy has become an important treatment for many malignant tumours after surgery and for palliative tumour care. Although modern radiotherapy technology is constantly improving, radiation damage to normal tissues is often difficult to avoid, and radiation-induced skin injury (RSI) is a common complication, manifested as skin erythema, peeling, ulceration, and even bone and deep organ damage, seriously affect the quality of life for patients. Basic research and clinical trials related to RSI have achieved certain results, while no researchers have conducted comprehensive bibliometric studies.

**Objective:**

A comprehensive bibliometric analysis of publications on RSI published between 2004 and 2023 was conducted to identify current hotspots and future directions in this area of study.

**Methods:**

RSI-related publications published between January 1, 2004, and December 31, 2023, were retrieved from the Web of Science Core Collection (WoSCC) database for analysis using VOSviewer and CiteSpace analytics.

**Results:**

A total of 1009 publications on RSI from 2004 to 2023 were included in the WoSCC database. The United States had the highest productivity with 299 papers, accounting for 29.63% of the total production, followed by China with 193 papers (19.13%) and Japan with 111 papers (11.00%). In terms of research institutions and journals, the University of Toronto and *Journal of Supportive Care in Cancer* published the highest number of papers. Professor Edward Chow published the most articles, while Professor Shuyu Zhang was the most cited. The top ten most-cited papers focused on the pathogenesis, prevention, and management of RSI. Keyword co-occurrence analysis and the top 25 keywords with the strongest citation bursts suggest that current research focuses on the pathogenesis, prevention, and treatment management of RSI.

**Conclusion:**

This study conducted a systematic bibliometric analysis of RSI publications from 2004 to 2023; identified the trends in RSI publications, major research countries, major research institutions, major research journals, major research authors, and major research keywords; and revealed the future development direction and research hotspots of this field. This study provides a valuable reference for future RSI research.

## 1 Introduction

**Figure 1:**
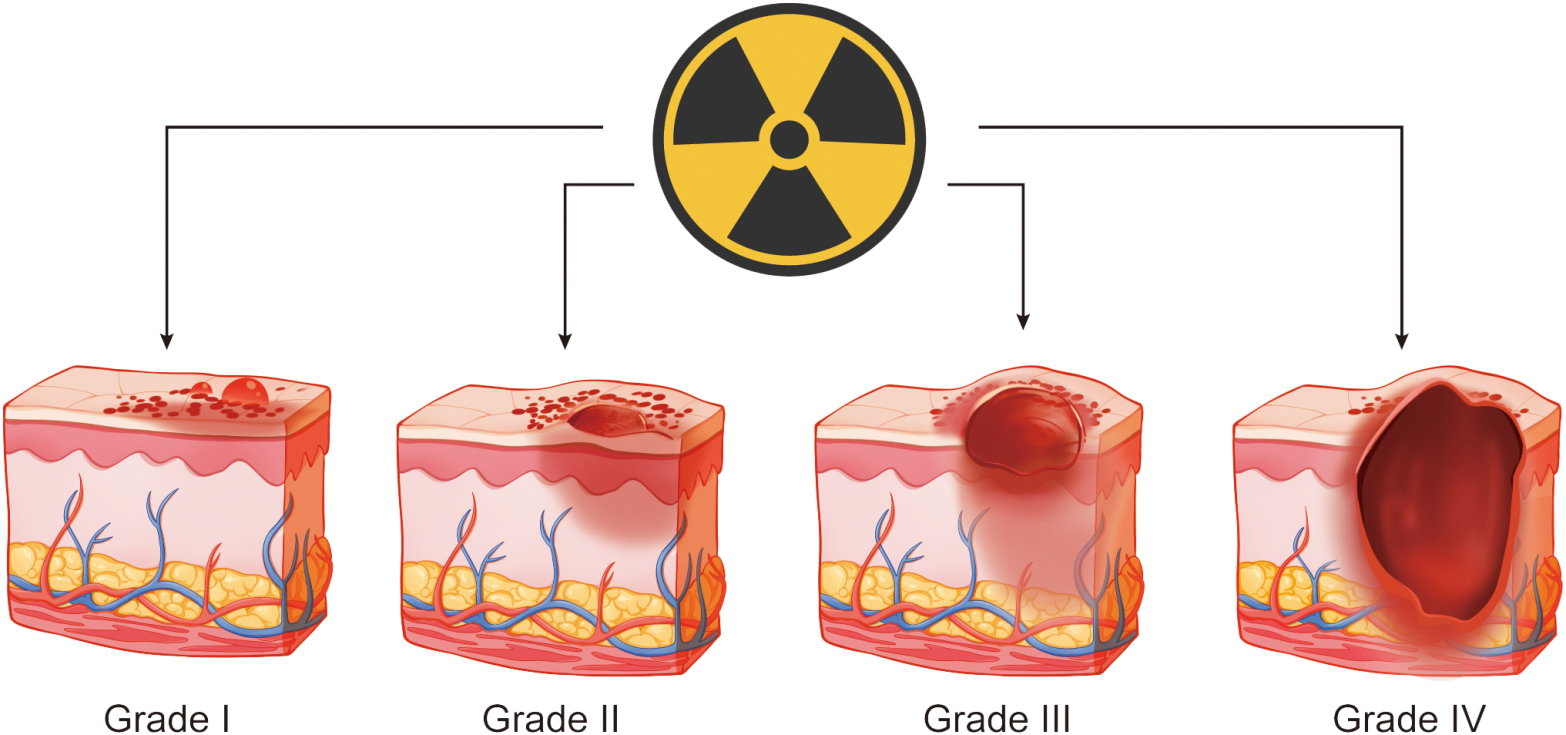
Schematic diagram of radiation-induced skin injury.

Radiation therapy has become an important treatment for many malignant tumours after surgery and for palliative tumour care (1). Over half of the patients with malignant tumours require radiation therapy (2). Although modern radiotherapy technology is constantly improving, radiation damage to normal tissues is often difficult to avoid, and radiation-induced skin injury (RSI) is a common complication. Approximately 95% of radiotherapy patients experience moderate to severe skin damage, resulting in skin erythema and peeling, and approximately 20%-25% of patients experience skin erosion and ulcers (**Figure 1**) (3–5). RSI caused by interventional therapy can also simultaneously occur (6). Owing to the cumulative effect of radiation on the skin, RSI is progressive, and complications can easily occur including secondary infection, aggravated tissue damage, ulcer wound deepening, and even bone and deep organ damage, which creates great difficulties in clinical treatment (7). With the increasing annual incidence of malignant tumours, RSI is gradually becoming the main factor affecting the quality of life and prognosis of patients with tumours (8).

However, no clear standard exists for the treatment of RSI, and the prevention and management of RSI in clinical practice are usually based on personal experience, while evidence-based treatments are lacking (9). Therefore, a comprehensive analysis of the global development status, research hotspots, and future development trends of RSI is immensely significant.

Bibliometrics is a scientific discipline that employs metering methods, including mathematics and statistics, to conduct comprehensive research on both quantitative and qualitative aspects of the literature (10). The analysis enables the identification of developmental laws and of research hotspots within the research field, as well as prediction of future directions and trends. VOSviewer and CiteSpace are two widely used bibliometric analysis tools for quantitatively describing and visually analysing literature (11). In recent years, numerous bibliometric analyses have been conducted in the fields of diabetes and hypertension; however, no such studies have been conducted on RSI (12, 13). Therefore, we employ bibliometric methodologies along with these two tools to systematically analyse RSI-related literature within the Web of Science Core Collection (WoSCC) database. The objective of this study is to explore the global research status quo and identify hotspots and frontiers in RSI research while predicting future development trends to provide a novel perspective for clinical prevention and treatment.

## 2 Materials and methods

### 2.1 Database collection and search strategies

**Figure 2:**
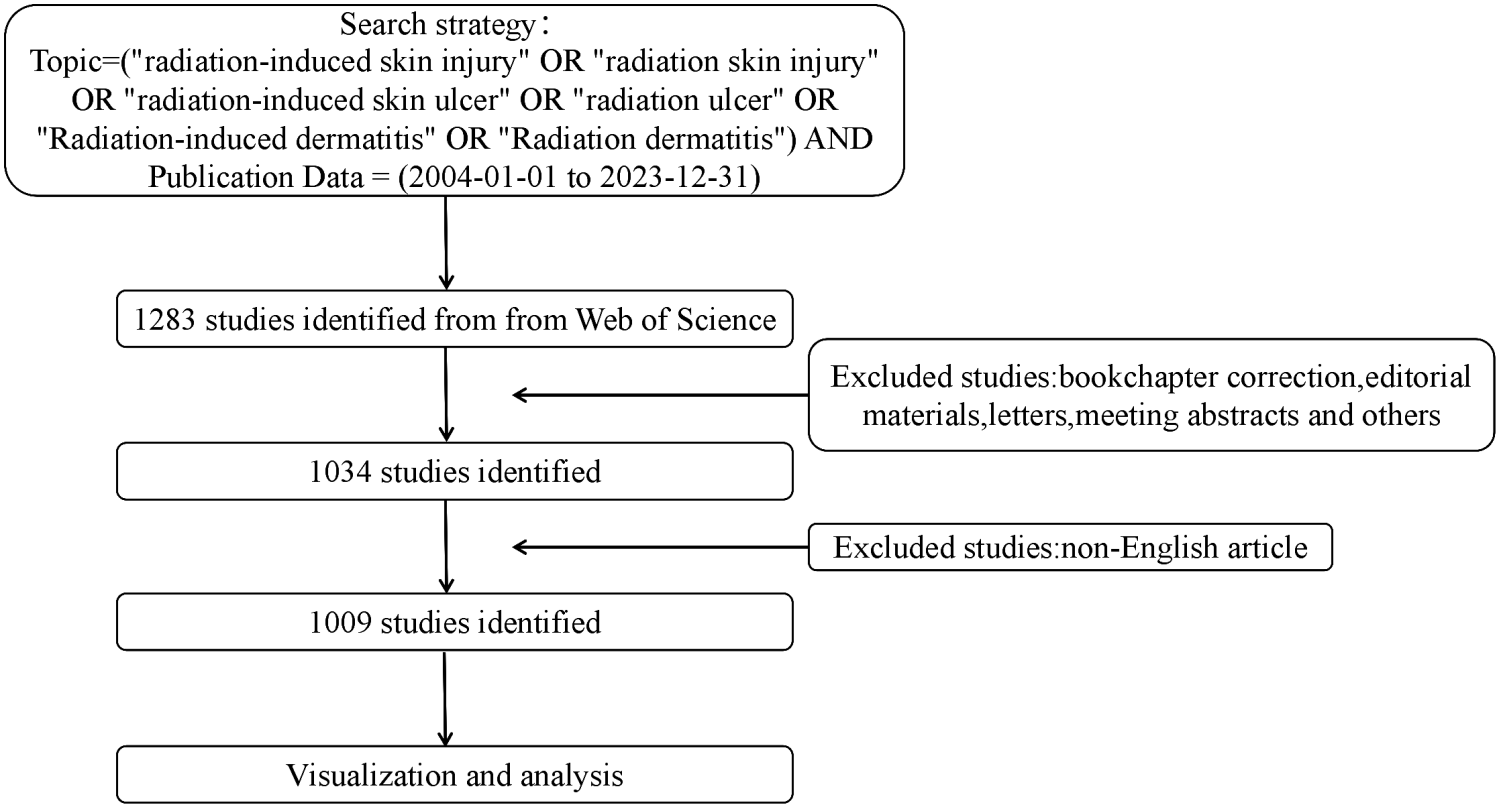
Research screening flow chart.

We searched for RSI-related literature between 2004 and 2023 collected from the WoSCC database. The following retrieval formula was used: Topic = (“radiation-induced skin injury” OR “radiation skin injury” OR “radiation-induced skin ulcer” OR “radiation ulcer” OR “Radiation-induced dermatitis” OR “Radiation dermatitis”) AND Publication Data = (2004-01-01 to 2023-12-31). First, 1283 papers were retrieved. After excluding conference abstracts, letters, and other types of papers, as well as non-English literature, a total of 1,009 papers were included in the analysis. The information gathered from the selected articles encompassed the annual count of publications and citations, contributing authors, their affiliations, the year of publication, originating country, publishing journal, utilized keywords, and the H-index. **Figure 2** shows the search keywords and strategies used.

### 2.2 Bibliometric analysis

The data were analysed using CiteSpace (6.2.4R Premium) and VOSviewer (1.6.18), which was created by Waltman et al. in 2009 and is a Java-based free software for analysing large amounts of documentary data and displaying them in map format. The results of research in a certain field can then be visualised by mapping literature co-citation networks (14). Professor Chaomei Chen created CiteSpace software, which envisions the use of an experimental framework to investigate new concepts and evaluate existing techniques (15). This enables users to better understand knowledge areas, research frontiers and trends, and predict their future research progress. The number of publication reflects the research heat of the field, while the number of citations reflects the influence of the publication in the academic community.

## 3 Results

### 3.1 Overall trends of publications

**Figure 3:**
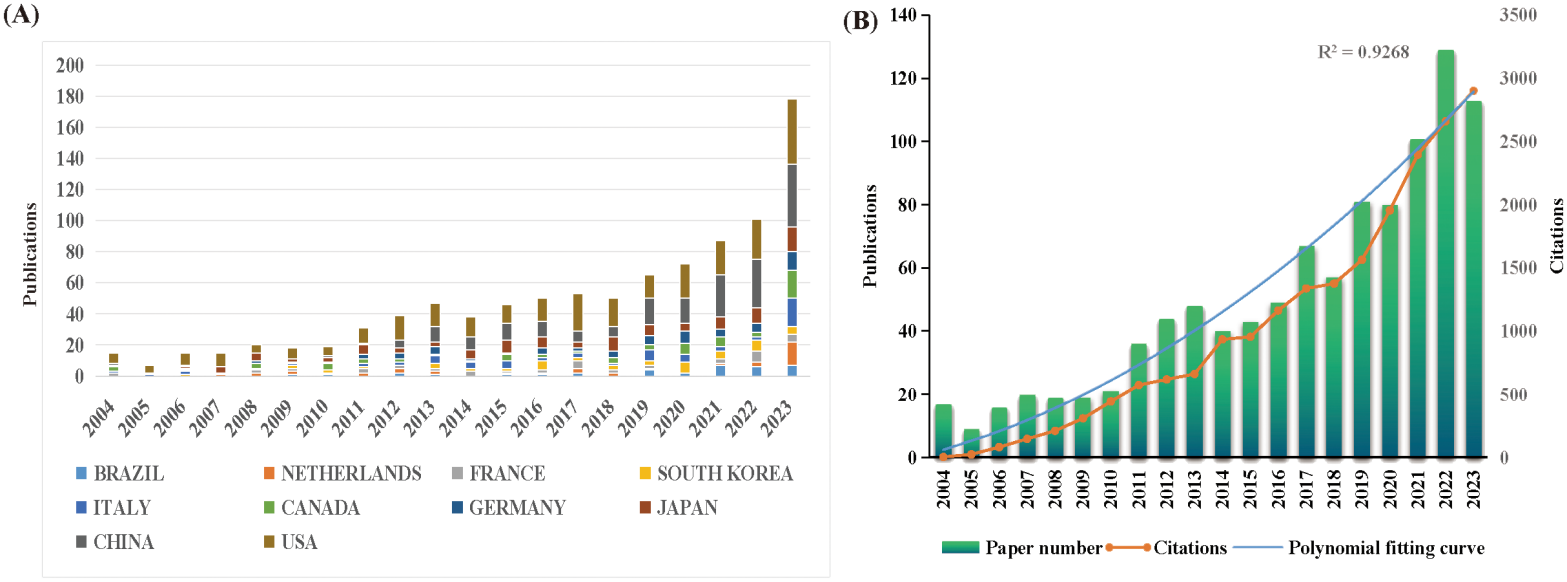
**(A)** The number of publications by country during the past 20 years. **(B)** The Number of annual publications and citations from 2004 to 2023.

In total, 1009 publications were included in this study, including 866 original articles and 143 review articles. Among the 1009 articles, the top three producing countries are the United States (= 299,29.63%), China (= 193,19.13%), and Japan (= 111,11.00%). Together, these three countries contributed 59.76% of the publications in the RSI field (**Figure 3A**). Over the past 20 years, the number of RSI-related publications per year has gradually increased globally from 17 publications in 2004 to 113 in 2023, reaching a peak of 129 in 2022. A strong relationship exists between the number of citations and the year of publication (R^2^ = 0.9268) (**Figure 3B**). These results indicate that RSI has attracted the attention of researchers worldwide.

### 3.2 Analysis of global publication distribution

**Figure 4:**
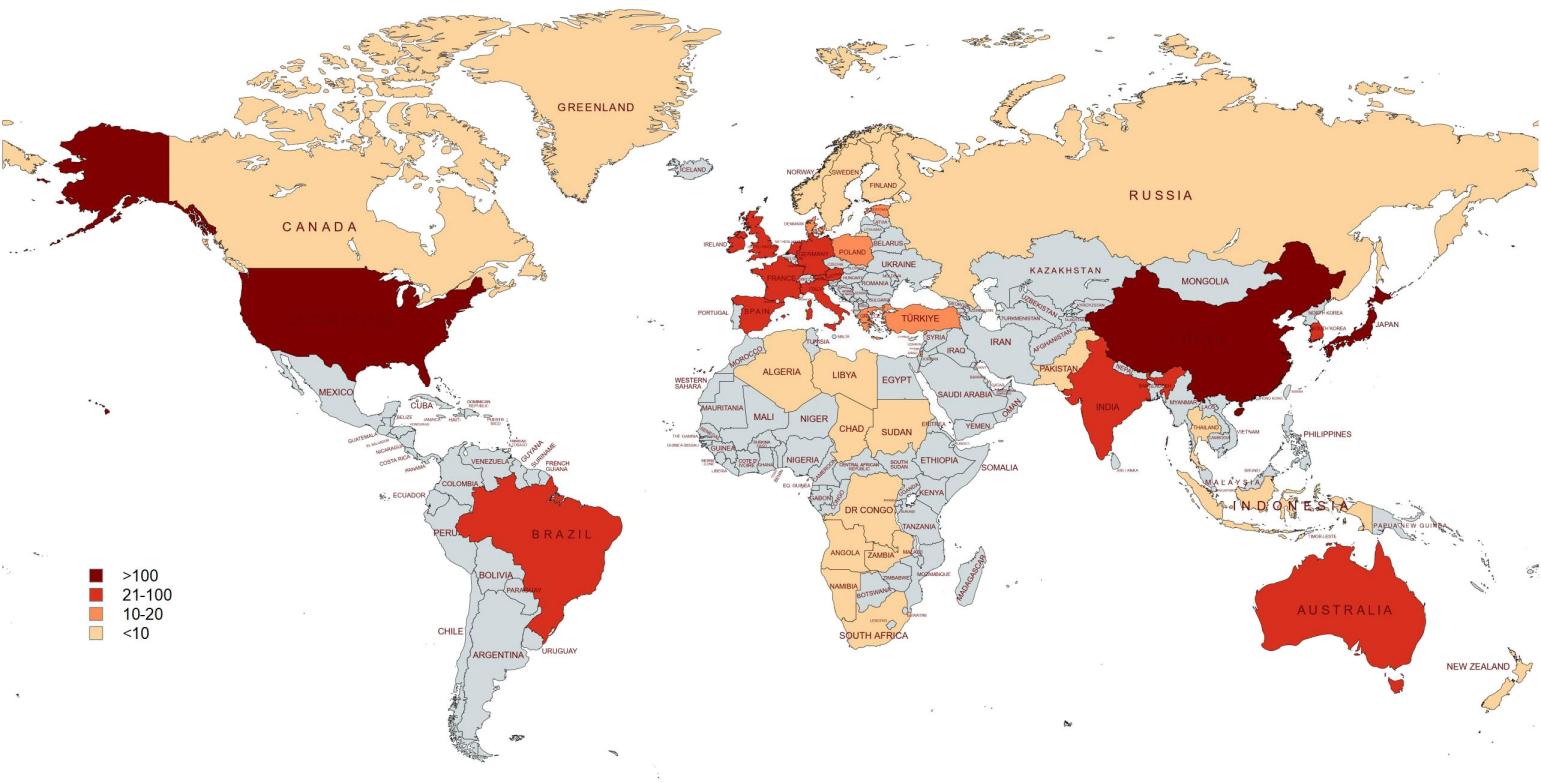
Visualization of the global distribution of RSI publications.

**Figure 5:**
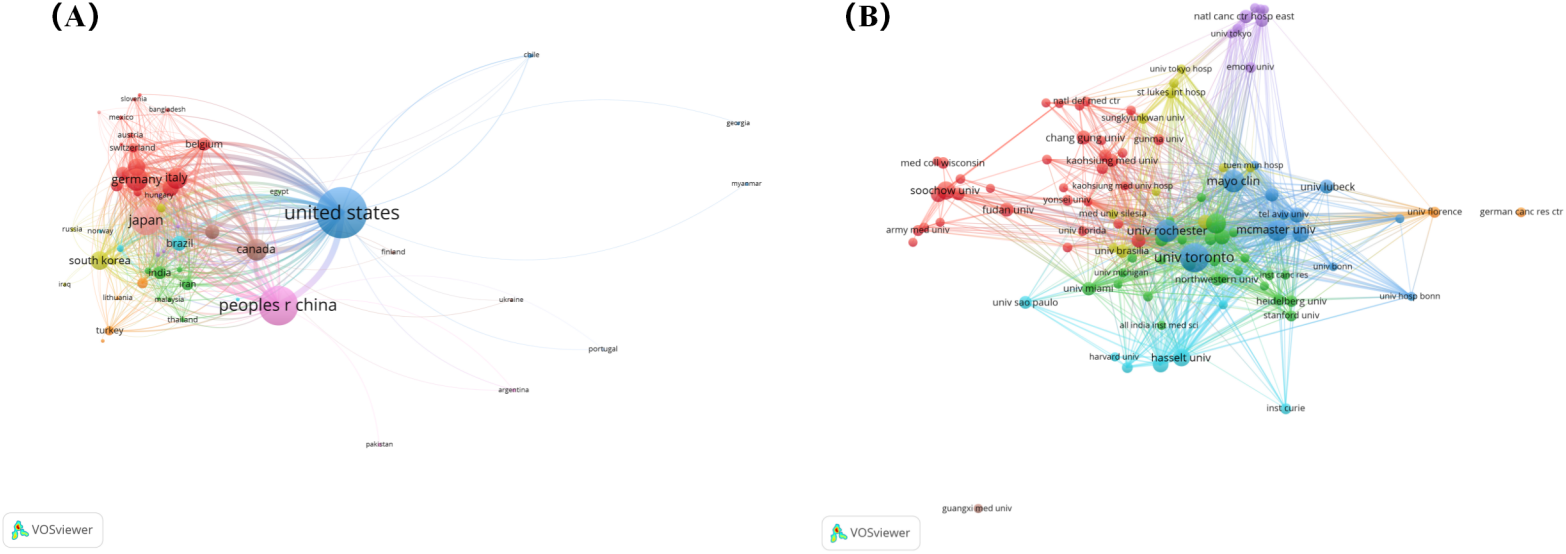
**(A)** Network of RSI research international cooperation. **(B)** Visualisation network of RSI research institution collaboration.

Over the past 20 years, RSI research has been published in 66 countries and regions worldwide. The global distribution of RSI publications is shown in **Figure 4**. The top ten countries with the largest number of publications are the United States, China, Japan, Germany, Canada, Italy, South Korea, France, the Netherlands, and Brazil, with the top three countries accounting for more than half of the total (**Table 1**). The United States has the highest number of references, up to 9694, which is significantly ahead of other countries. In addition, we analysed global RSI research international partnerships. As shown in **Figure 5A**, international cooperation is closely linked, especially between China and the United States. Countries that publish fewer publications have less international cooperation.

**Table 1:** Top 10 countries with the highest number.

Rank	Country	Publications	Citations	%of (1009)
1	the United States	299	9694	29.63%
2	China	193	3045	19.13%
3	Japan	111	1586	11.00%
4	Germany	69	1499	6.84%
5	Canada	67	2455	6.64%
6	Italy	65	1911	6.44%
7	South Korea	51	468	5.05%
8	France	47	2179	4.66%
9	Netherlands	40	1389	3.96%
10	Brazil	35	709	3.47%

### 3.3 Analysis of institutional distribution

The top ten institutions with the highest publication volume are distributed across six different countries: Canada, the United States, China, Belgium, the Netherlands, and Italy (**Table 2**). The University of Toronto published the most papers, followed by the Mayo Clinic and University of Rochester. In addition, two cancer institutions are present in the mix: the Memorial Sloan Kettering Cancer Center and the Netherlands Comprehensive Cancer Organisation. To further analyse the collaboration between different institutions, we used VOSviewer to map the RSI research institution collaboration visualisation network (**Figure 5B**).

**Table 2:** Top 10 institutions with the highest volume of publications.

Rank	Institution	Country	Publications	Citations	%of (1009)
1	University of Toronto	Canada	38	737	3.77%
2	Mayo clinic	USA	24	399	2.38%
3	**University of Rochester**	USA	24	798	2.38%
4	McMaster University	Canada	21	217	2.08%
5	Memorial Sloan Kettering Cancer Center	USA	20	659	1.98%
6	Soochow University	China	17	285	1.68%
7	Hasselt University	Belgium	16	237	1.59%
8	Netherlands comprehensive cancer organisation	Netherlands	14	73	1.39%
9	Jessa hospital	Belgium	13	216	1.29%
10	Azienda Ospedaliero-Universitaria Careggi	Italy	12	72	1.19%

### 3.4 Analysis of journal distribution

**Figure 6:**
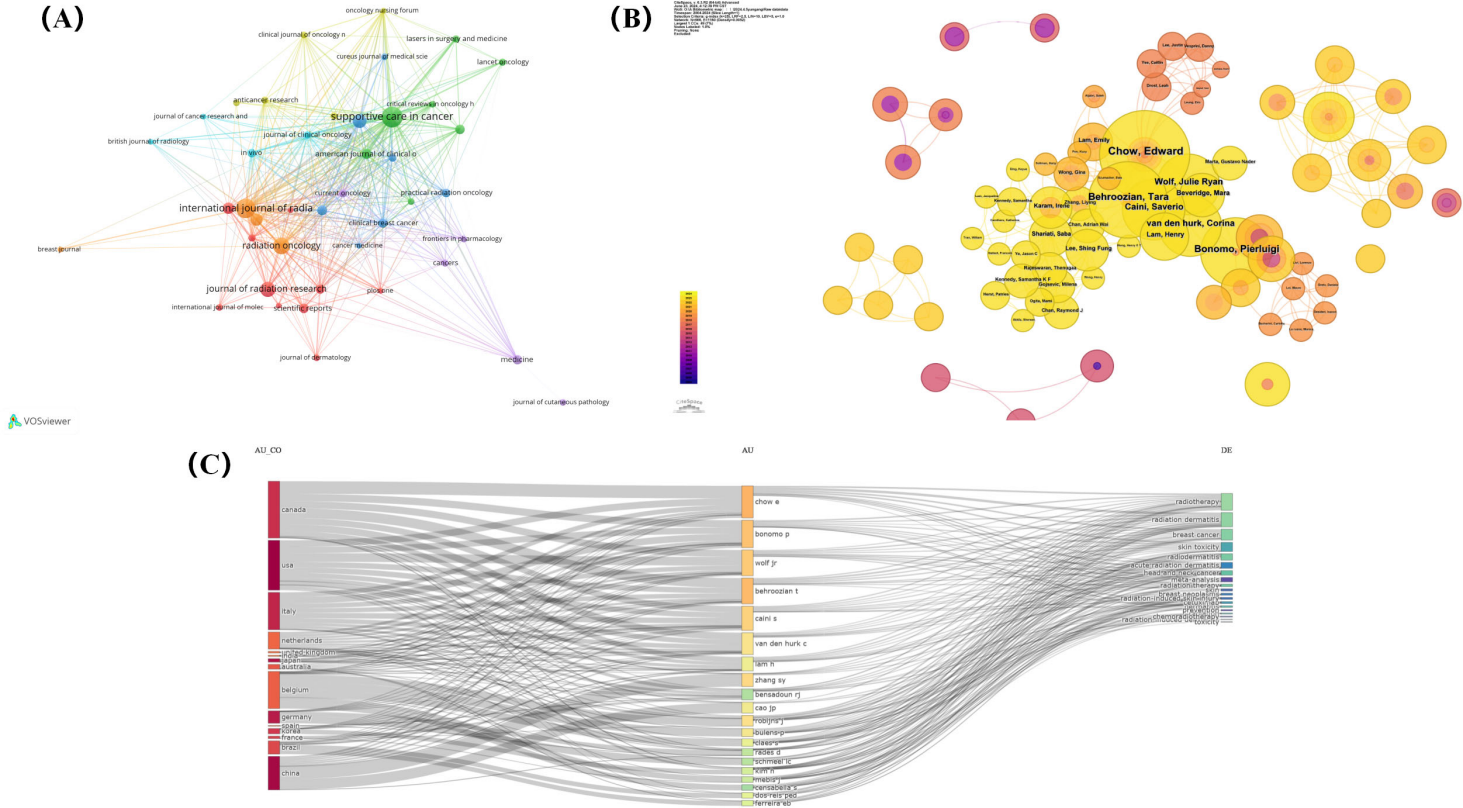
**(A)** Co-citation network visualisation of journals. **(B)** Co-citation network visualisation of authors. **(C)**Country, author and keywords Sankey diagram.

*Supportive Care in Cancer*, a leading publisher of RSI research, was followed by the *International Journal of Radiation Oncology Biology Physics* and *Radiation Oncology* (**Table 3**). From 2004 to 2023, the top ten journals with the most publications accounted for 22.50% of the total. In addition, we used co-citation analysis to further study the cooperative relationships between journals (**Figure 6A**).

**Table 3:** Top 10 journals with the highest volume of publications.

Rank	Journals	Publications	Citations	%of (1009)
1	*Supportive Care in Cancer*	44	1162	4.36%
2	*International Journal of Radiation Oncology Biology Physics*	39	1221	3.87%
3	*Radiation Oncology*	29	557	2.87%
4	*Journal of Radiation Research*	25	325	2.48%
5	*Radiotherapy and Oncology*	22	627	2.18%
6	*Frontiers in Oncology*	18	202	1.78%
7	*Radiation Research*	15	343	1.49%
8	*American Journal of Clinical Oncology-Cancer Clinical Trials*	12	398	1.19%
9	*BMC Cancer*	12	331	1.19%
10	*Medicine*	11	91	1.09%

### 3.5 Analysis of authors distribution

The 10 authors who have published the most in the field of RSI worldwide from six different countries, published 164 articles, representing 16.25% of all publications in the last 20 years (**Table 4**). Professor Edward Chow from the University of Toronto is the most prolific author in the field, followed by Prof. Pierluigi Bonomo from Azienda Ospedaliero-Universitaria Careggi, and Prof. Tara Behroozian from McMaster University. The most cited author is Shuyu Zhang, a professor at Sichuan University, China (**Figure 6B**). In addition, although Professor Jianping Cao from Soochow University in China has the lowest number of publications among the top 10, his citation counts are only lower than those of Shuyu Zhang and Edward Chow (**Figure 6C**). Hence, the achievements of Chinese scholars in the field of RSI have been recognized by scholars worldwide.

**Table 4:** Top 10 authors with the highest volume of publications.

Rank	Author	Country	Publications	Citations	%of (1009)
1	Chow, Edward	Canada	28	280	2.78%
2	Bonomo, Pierluigi	Italy	20	142	1.98%
3	Behroozian, Tara	Canada	19	106	1.88%
4	Wolf, Julie Ryan	USA	16	127	1.59%
5	Caini, Saverio	Italy	15	82	1.49%
6	Robijns,Jolien	Belgium	14	203	1.39%
7	Van den Hurk, Corina	Netherlands	14	73	1.39%
8	Zhang, Shuyu	China	14	283	1.39%
9	Bulens, Paul	Belgium	12	201	1.19%
10	Cao, Jianping	China	12	267	1.19%

### 3.6 Analysis of top 10 citations articles

Of the top 10 cited articles, the most-cited is ‘A multicentre randomised trial of breast intensity-modulated radiation therapy to reduce acute radiation dermatitis’ published by Professor Jean-Philippe Pignol, with as many as 552 citations. The lowest is Professor Lynda D. Woodruff’s ‘The efficacy of laser therapy in wound repair: A meta-analysis of the literature’, which has been cited 249 times (**Table 5**). These articles are mainly concerned with RSI prevention and treatment management.

**Table 5:** Top 10 articles with the highest number of citations.

Rank	Title	Journal	Author	Year	Citations
1	A multicentre randomised trial of breast intensity-modulated radiation therapy to reduce acute radiation dermatitis	*J Clin Oncol*	Pignol, JP	2008	552
2	Radiation dermatitis: Clinical presentation, pathophysiology, and treatment 2006	*J Am Acad Dermatol*	Hymes, SR	2006	406
3	Low-level laser therapy for wound healing: Mechanism and efficacy	*Dermatol Surg*	Posten, W	2005	391
4	Ionizing Radiation: The Good, the Bad, and the Ugly	*J Invest Dermatol*	Ryan, JL	2012	325
5	Clinical practice guidelines for the prevention and treatment of EGFR inhibitor-associated dermatologic toxicities	*Support Care Cancer*	Lacouture, ME	2011	324
6	Angiographic Success and Procedural Complications in Patients Undergoing Percutaneous Coronary Chronic Total Occlusion Interventions A Weighted Meta-Analysis of 18,061 Patients From 65 Studies	*JACC Cardiovasc Interv*	Patel, VG	2017	275
7	NBTXR3, a first-in-class radioenhancer hafnium oxide nanoparticle, plus radiotherapy versus radiotherapy alone in patients with locally advanced soft-tissue sarcoma (Act. In. Sarc): a multicentre, phase 2-3, randomised, controlled trial	*Lancet Ocol*	Bonvalot, S	2019	273
8	The Efficacy of Low-Power Lasers in Tissue Repair and Pain Control: A Meta-Analysis Study	*Photomed Laser Surg*	Enwemeka, CS	2004	268
9	Prophylaxis and management of acute radiation-induced skin reactions: a systematic review of the literature	*Curr Oncol*	Salvo, N	2010	258
10	The efficacy of laser therapy in wound repair: A meta-analysis of the literature	*Photomed Laser Surg*	Woodruff, LD	2004	249

**Figure 7:**
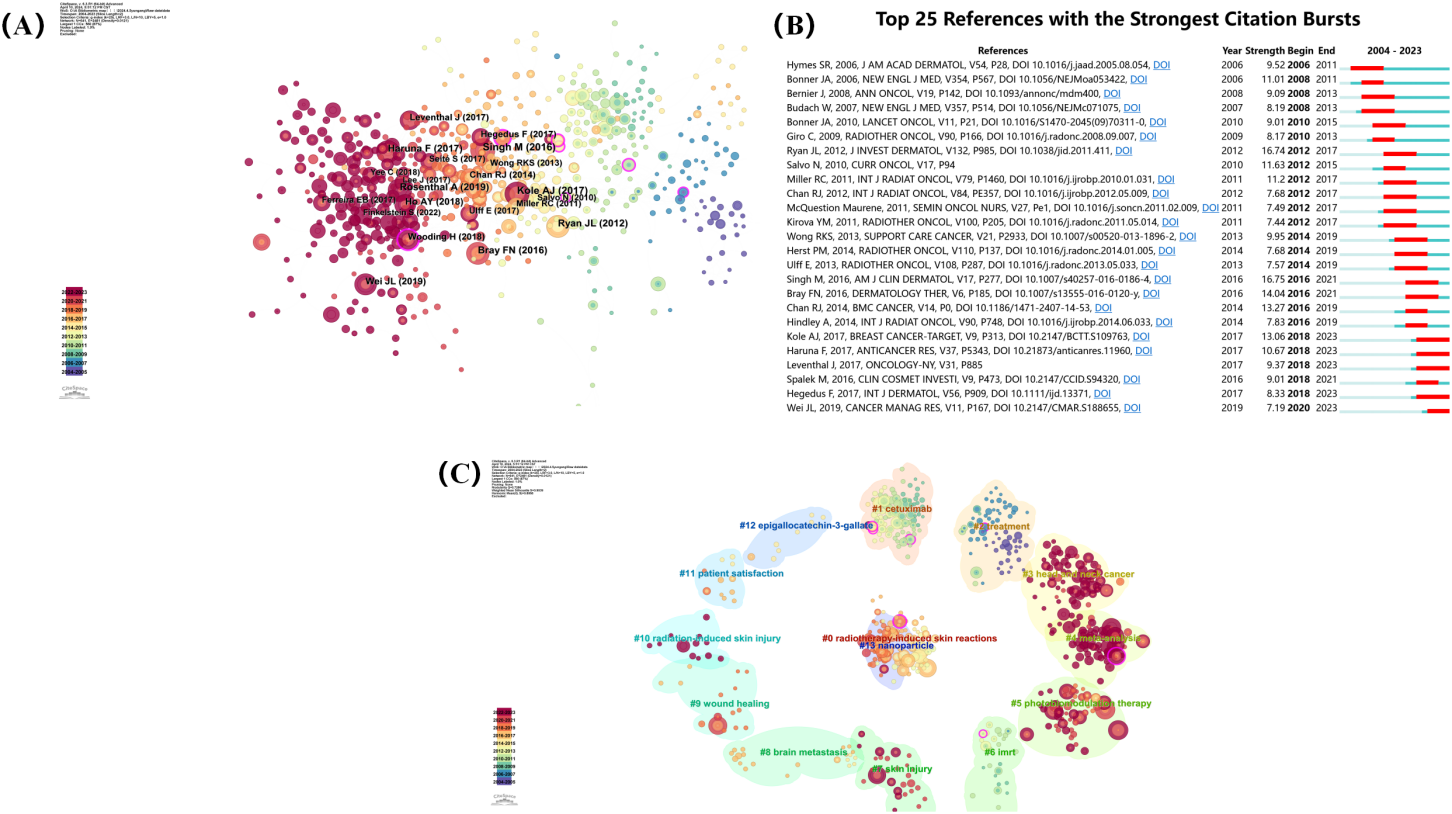
**(A)** Co-citation network visualization of references. **(B)** Top 25 references with the strongest citation bursts. **(C)** Co-citation references topic analysis.

CiteSpace software was used to generate a co-citation network diagram depicting the relationships among the references (**Figure 7A**). The articles with the highest co-citation frequency include Manni Singh’s ‘Radiodermatitis: A Review of Our Current ‘Understanding’, Julie L Ryan’s ‘Ionizing radiation: the good, the bad, and the ugly’, and Fleta N Bray’s publication titled ‘Acute and Chronic Cutaneous Reactions to Ionizing Radiation Therapy’ (3, 16, 17). These three reviews comprehensively describe the pathogenesis, clinical manifestations, differential diagnosis, prevention, and treatment of skin injuries caused by ionising radiation.

A reference citation burst refers to a phenomenon in which the number of citations surges after an article is published. This indicates that scholars have paid significant attention to related topics. We analysed the top 25 references with the strongest citation bursts over the past 20 years using CiteSpace’s burst detection function (**Figure 7B**). In addition, we divided the reference co-citation titles into 14 topics. These are ‘radiotherapy-induced skin reactions’, ‘cetuximab’, ‘treatment’, ‘head and neck cancer’, ‘meta-analysis’, ‘photobiomodulation therapy’, ‘IMRT’, ‘skin injury’, ‘brain metastasis’, ‘wound healing’, ‘radiation-induced skin injury’, ‘patient satisfaction’, ‘epigallocatechin-3-gallate’, and ‘nanoparticle’ (**Figure 7C**). Research on RSI has mainly focused on radiotherapy-induced skin reactions and treatment after skin injury.

**Figure 8:**
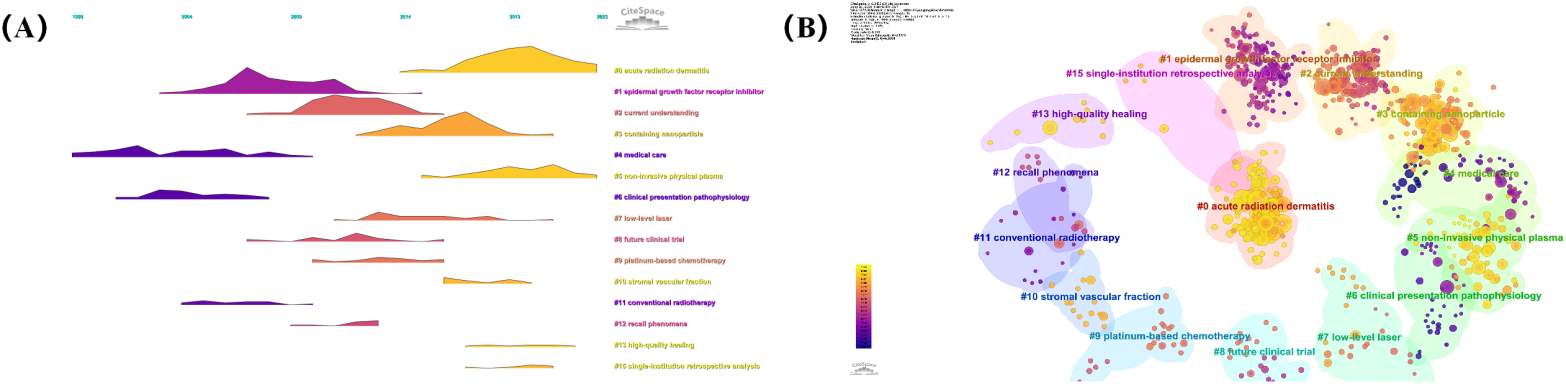
**(A)** Volcano map of co-cited references. **(B)** Cluster map of co-cited references.

In addition, we also conducted co-cited reference clustering and temporal clustering analysis (**Figure 8**). We found that medical care (cluster 4) and clinical presentation pathophysiology (cluster 6) were early research hotspots. epidermal growth factor receptor inhibitor (cluster 1), current understanding (cluster 2), low-level laser (cluster 7), future clinical trial (cluster 8), Platinate-based chemotherapy (cluster 9), conventional radiotherapy (cluster 11), recall phenomena (cluster 12) is a research hotspot in the middle period. acute radiation dermatitis (cluster 0), containing nanoparticle (cluste 3), non-invasive physical plasma (cluster 5), stromal vascular fraction (cluster10), high-quality healing (cluster 13), single-institution retrospective analysis (cluster 15) is a hot topic and trend in the field.

### 3.7 Analysis of RSI field frontiers and hotspots

**Figure 9:**
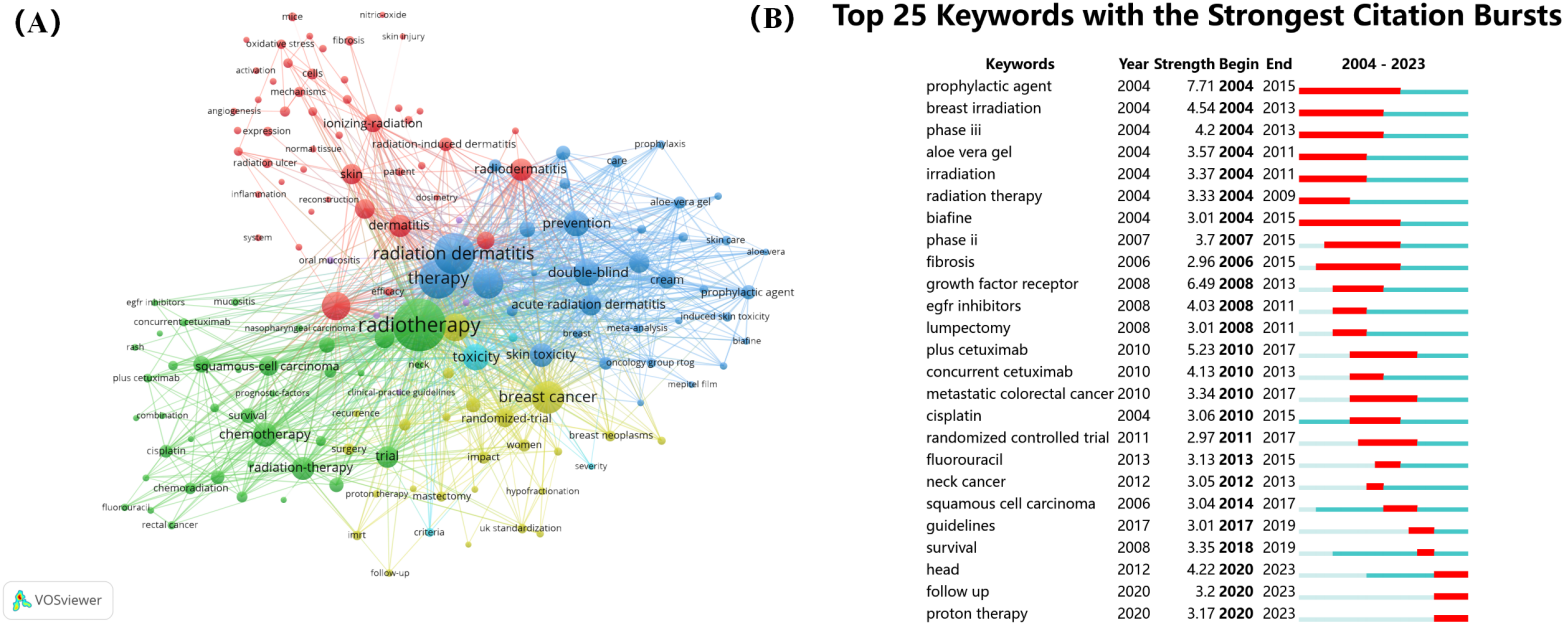
**(A)** Co-occurrence network visualization of keywords. **(B)** Top 25 keywords with the strongest citation bursts.

Keyword co-occurrence analysis can identify current research hotspots in the field of RSI, which is of great significance for the development of future research trends. By drawing a co-occurrence network of keywords, we divided them into four categories (**Figure 9A**).

**Figure 10:**
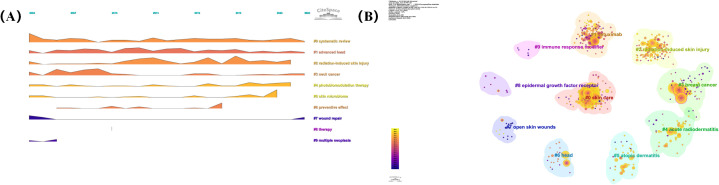
**(A)** Volcano map of keywords. **(B)** Cluster map of keywords.

Cluster 1 (indicated in green) mainly involves radiotherapy, Cluster 2 (indicated in blue) mainly focuses on radiation dermatitis therapy, Cluster 3 (indicated in red) mainly focuses on dermatitis, and Cluster 4 (indicated in red) mainly focuses on breast cancer. In addition, we analysed the top 25 keywords with the strongest citation bursts over the last 20 years (**Figure 9B**). These results indicate that the current research focus is still on RSI prevention and treatment, mainly including growth factors, aloe vera gel, and related randomised controlled trials, among which the most common causes are radiotherapy for breast cancer and head and neck cancer. In order to more directly display the change of research hotspot over time, we drew the keyword clustering and time clustering analysis volcano map through CiteSpace (**Figure 10**).

**Figure 11:**
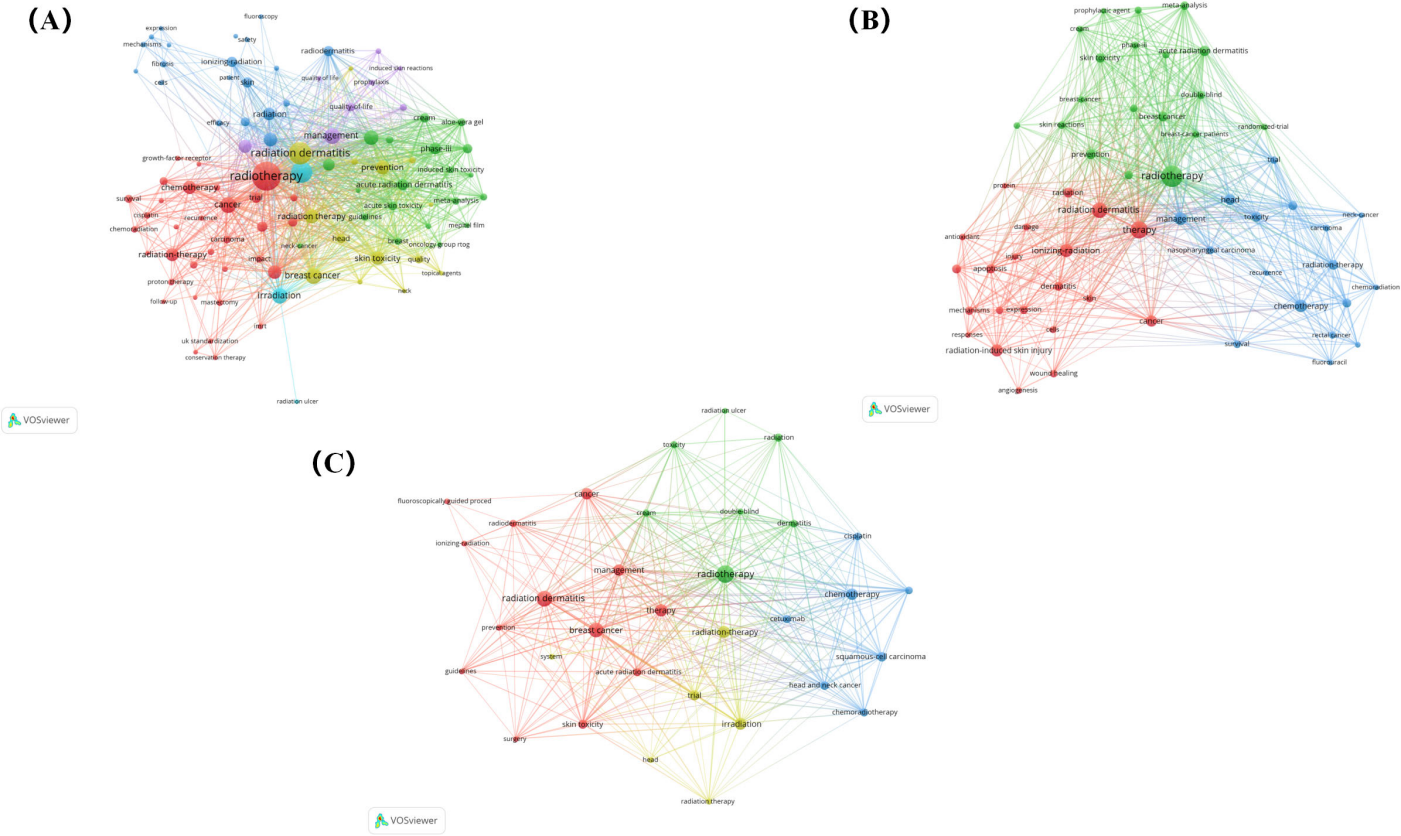
**(A)** Co-occurrence network visualization of keywords in the United States. **(B)** Co-occurrence network visualization of keywords in China. **(C)** Co-occurrence network visualization of keywords in Japan.

To further analyse the differences in research hotspots among different countries, we compare the top three countries with the largest volume of publications: the United States, China, and Japan (**Figure 11**). We found that the three countries re very close to each other in terms of research focus, mainly covering the pathogenesis, prevention, and treatment of RSI. However, the United States has paid more attention to the pathogenesis of RSI, such as radiation dermatitis and skin toxicity. China focuses on RSI prevention and wound healing mechanisms such as antioxidants and angiogenesis. However, Japan has been paying more attention to RSI treatments.

## 4 Discussion

Bibliometrics is often used to evaluate the research output and influence of researchers, institutions, and countries in a certain field, as well as changing trends in the research field. This helps researchers understand the current research hotspots and future development directions. In this study, we analysed 866 original articles and 143 review articles related to RSI from 2004 to 2023, showing an overall upward trend in the number of publications and citations per year. This indicates that global understanding of RSI is gradually increasing, which may be related to an increase in the number of patients with malignant tumours requiring radiation therapy. The United States leads in both the number of publications and citations in the field of RSI, far exceeding other countries. At the same time, among the top 10 institutions with the highest number of publications, three are from the United States, which shows that this country is in a leading position in research in this field. The most widely published RSI journal is *Supportive Care in Cancer*. This journal deals primarily with interventions to reduce symptoms and side effects in patients with cancer throughout their lives. The journal with the highest number of RSI citations is the *International Journal of Radiation Oncology Biology Physics*, which is mainly publishes articles concerning radiation oncology, radiobiology, medical physics, and original laboratory and clinical research related to education and health policies in this field. In the field of RSI, the most published author is Professor Edward Chow from the Department of Radiation Oncology at the University of Toronto in Canada, while the most cited author is Professor Shuyu Zhang from the School of Basic Medicine of Sichuan University, China. Hence, Canadian and Chinese scholars have conducted in-depth research in this field and have been recognized by domestic scholars. The top ten most-cited articles predominantly focused on the pathogenesis, treatment, and prevention of RSI. This is the current research hotspot and future focus of RSI.

### 4.1 RSI pathogenesis

Skin injuries caused by radiation include acute and chronic radiation dermatitis. The pathogenic mechanisms include oxidative stress, inflammatory fibrosis, and cell senescence.

#### 4.1.1 Oxidative stress reaction

When tissues are exposed to ionizing radiation, water molecules in the skin ionise and produce large amounts of reactive oxygen species (ROS). ROS can bind to lipid molecules in the cell membrane and cause lipid peroxidation, which destroys cell structure and function (18). In addition, ionising radiation can directly damage cell DNA, causing DNA strand breaks and producing ROS, resulting in persistent oxidative stress that affects the repair of the DNA double-helix structure, resulting in cell function impairment and cell necrosis (19). Continuous oxidative stress hinders basal cell division, proliferation, migration to the surface, and keratinisation (20). Oxidative stress causes vascular endothelial cell damage, resulting in dermal microvascular occlusion, ischaemia, and hypoxia (21).

#### 4.1.2 Inflammatory fibrosis

After the tissue is exposed to ionising radiation, neutrophils are recruited to the irradiated site to release inflammatory mediators and stimulate an inflammatory response. Simultaneously, DNA damage, cell death, and ROS production further aggravate the inflammatory response (22). The acute inflammatory response is primarily triggered by pro-inflammatory cytokines [such as interleukin (IL)-1, IL-3, IL-5, IL-6, tumour necrosis factor-alpha (TNF-α)], chemokines (such as IL-3, eosinophil chemotactic factor), tyrosine kinase receptors, and adhesion molecules (ICAM-1, VCAM) (23). The chronic inflammatory phase predominantly involves macrophages, fibroblasts, and epithelial cells secreting transforming growth factor-beta (TGF-β) (24). TGF-β, a pro-fibrotic factor, stimulates fibroblasts to differentiate into myofibroblasts post-radiation, leading to an overproduction of extracellular matrix components such as collagen and fibronectin (25). TGF-β also reduces matrix metalloproteinase (MMP) activity in fibroblasts, affecting the degradation of the extracellular matrix (26). Accumulation of the extracellular matrix leads to increased skin tissue hardness and thickness, resulting in fibrosis. Skin fibrosis is a characteristic manifestation of chronic radiative injury. Fibrosis disrupts lymphatic and local blood circulation, reduces tissue perfusion, exacerbates the quality and functional impairment of post-radiation skin, and causes skin injuries that hinder wound healing.

#### 4.1.3 Cell senescence

Cell senescence is an evolutionarily conserved state of stable replication stagnation induced by pro-aging stressors. ROS and DNA damage caused by ionising radiation lead to cell cycle arrest and induce cell senescence (27). Senescent cells typically promote the secretion of a range of pro-inflammatory cytokines, growth factors, chemokines, and matrix metalloproteinases, collectively known as the senescence-associated secretory phenotype (SASP), and can induce tissue dysfunction via paracrine means (28). Chen et al. (29) found that in an animal model of RSI, the number of senescent cells gradually increased with the progression of radiation damage, and exogenous injection of senescent cells significantly promoted the progression of RSI in a rat model, indicating that senescent cells play a key role in the progression of RSI. Wang et al. (30) confirmed that cordycepin inhibited cell senescence in rat radiation ulcers and reduced the progression of radiation damage. Similarly, the removal of senescent cells can alleviate RSI. Dasatinib plus quercetin can selectively remove senescent cells by inducing apoptosis, reducing DNA damage, and maintaining the proliferative ability of tissue cells, thus preventing the development of RSI (31). Therefore, inhibiting cell senescence or removing senescent cells is expected to be a new strategy for reducing RSI.

At present, the pathogenesis of RSI is still studied at the cellular and molecular levels, but further studies are needed on the genes related to susceptibility to radioactive skin injury, the effects of radiation injury on epigenetic modification, and the interaction between immune response and microenvironment. By continuously improving the understanding of the pathogenesis, finding biomarkers for early prediction and diagnosis of RSI, and providing basis for personalized precision treatment, it is expected to become the main treatment strategy in the future.

### 4.2 RSI risk factors

Skin damage can occur in any part of the body after exposure to radiation and is more common in moist areas of the body and folds of the skin, such as the head and neck, mammary glands, armpits, perineum, and groin. RSI is common in patients with head and neck tumours, breast cancer, lung cancer, and sarcoma because of the higher radiation doses received by the skin. Risk factors for RSI mainly include patient-related and treatment-related factors.

#### 4.2.1 Patient-related factors

The anterior neck area, limbs, chest, abdomen, facial skin, and scalp are sensitive to radiation and are at a higher risk of RSI (32). Obesity, malnutrition, chronic sun exposure, and smoking may increase the risk of RSI (33). Patients with genetic disorders associated with impaired DNA repair, such as ataxic capillary dilatation, Bloom syndrome, Fanconi anaemia, Gorlin syndrome, and xeroderma chromatica, are at a higher risk of RSI (21).

#### 4.2.2 Treatment-related factors

The total dose of radiotherapy, dose/number of splits, irradiation volume, and skin surface area affect the degree of skin damage (34). Radiotherapy techniques (intensity-modulated radiotherapy and conventional two-dimensional radiotherapy), ray particle type, and energy also affect skin toxicity. Intensity-modulated radiotherapy (IMRT) can protect normal tissues and reduce the dose and volume of skin exposure, and the dose distribution is more uniform than that in traditional two-dimensional radiotherapy technology, which can reduce the hot spots of high doses to the skin, effectively reducing the incidence of acute wet peeling reactions and late reactions such as telangiectasia (17). Both concurrent and adjuvant chemotherapy can increase skin toxicity associated with radiotherapy. Traditional chemotherapy drugs (such as doxorubicin, methotrexate, fluorouracil, and bleomycin), paclitaxel, and epidermal growth factor receptor inhibitors increase the risk of RSI (35).

### 4.3 RSI prevention

The initial symptoms of RSI are mild, and with the accumulation of radiation in the skin, wet dermatitis appears, which can easily progress to chronic radioactive skin ulcers that do not heal and even cause deep tissue damage, such as radiation pneumonia and radiation cystitis (36, 37). Therefore, early prevention of RSI is very important. The main preventive measures against RSI include topical and systemic drugs, and modern radiotherapy techniques.

#### 4.3.1 Topical drugs

Topical corticosteroids are effective for preventing RSI. Regular use of topical corticosteroids during and weeks after radiotherapy may reduce the incidence of severe dermatitis, prevent severe radiation dermatitis, and reduce discomfort and itching (38). Shukla et al. (39) found that corticosteroids could effectively prevent radiation-induced skin toxicity. The incidence of wet peeling was 13% in the corticosteroid prevention group and 37% in the control group (without drug treatment). Although topical corticosteroids have shown some efficacy in RSI, there are some long-term limitations and potential side effects, including skin thinning, pigment changes, telangiectasia and drug dependence, and even local infections (40). Therefore, when selecting local topical drugs, short-term use should be tried to avoid long-term dependence; Choose low or medium acting corticosteroids to reduce the risk of side effects; Evaluate skin status regularly and adjust treatment regimen in time. Additionally, recombinant human granulocyte macrophage-stimulating factor and epidermal growth factor promote cell proliferation, reduce inflammation, improve skin barrier function and repair damaged skin (41). However, growth factor is relatively expensive, poor *in vitro* stability and easy to degrade, especially after long-term use of growth factor in the skin after radiotherapy, whether there is abnormal cell proliferation and increase the potential possibility of tumour needs further investigation (42). In general, growth factors have significant efficacy and multiple advantages in the prevention of radioactive skin injury, but at the same time, there are certain limitations. Future research is needed to further optimize the stability and application methods of growth factors, reduce costs, and increase their clinical penetration. At the same time, more clinical studies are needed to evaluate the long-term safety and efficacy of growth factors. Silicone film-forming gel dressings, silver ion dressings, and triethanolamine also play vital roles in preventing radiation dermatitis (43).

#### 4.3.2 Systemic drugs

Oral pentoxifylline is beneficial in reducing delayed skin changes, such as skin fibrosis and necrosis, after radiation therapy; however, the preventive effect against acute radiation dermatitis is similar to that of placebo (44). Small randomised trials have evaluated egg white hydrolytic enzymes, antioxidants, zinc supplements, sulfoalumin, and gingerin, but few have documented their effectiveness in whole-body prevention (45, 46). Therefore, full-body medications are not recommended to reduce RSI.

#### 4.3.3 Modern radiotherapy techniques

The continuous development and improvement of modern radiotherapy technology are fundamental measures to prevent RSI. Precision radiotherapy is gradually replacing traditional radiotherapy techniques. IMRT and volumetric modulated arc therapy (VMAT) use precise positioning, planning, and illumination formulas, which can significantly reduce the radiation of normal tissues outside the target area and reduce the incidence of skin reactions (47). The use of hyperfractionation radiotherapy for the treatment of milk adenocarcinoma is increasing. Raising the total equal-effect dose with a higher fractional dose in a short course of therapy, compared with standard fractionation therapy, can reduce acute radiative dermatitis, such as diamantus, the incidence of itching, pigmentation, and pain (48).

### 4.4 RSI therapy

During radiation therapy, the skin at the site of radiation should be evaluated at least once a week, and the colour, texture, and sensation of the skin should be carefully observed. The therapeutic management of RSI is guided by the severity of skin injury, and treatment depends on the severity of the skin injury.

#### 4.4.1 Acute radiation dermatitis

Grade I radiation dermatitis is characterised by mild erythema and dry peeling, and generally requires application of a moisturiser. Low- and medium-efficiency topical steroid hormones can be used (49). Grade II–III radiation dermatitis is characterised by wet peeling of the skin folds or other parts of the skin. Treatment includes prevention of secondary skin staining and promotion of wet peeling healing. Silicone gel dressings, hydrogels, and hydrocolloidal dressings can keep the wound moist, promote wound healing, and effectively relieve dermatitis above grade II (9, 20). At present, there are many kinds of gel dressings, and how to choose the right dressing according to the type of radioactive skin injury is a problem that needs to be solved in clinical management. For wounds combined with infection, dressings containing antibacterial agents or silver ions can be selected to prevent infection and accelerate wound healing (50). However, the pathogenesis of radioactive skin injury is complex, and single-function dressings are not sufficient to meet clinical needs. The development of multifunctional dressings that can adapt to radioactive wounds will be the main research direction in the future. Co-infections should be treated with topical or systemic antibiotics. Grade IV radiation dermatitis may result in skin necrosis and refractory ulceration. Treatment methods mainly include surgical debridement, musculocutaneous flaps with abundant blood flow, and flap transplantation to cover the wound. Physical treatment should be based on individual conditions (22). Platelet-rich plasma, hyperbaric oxygen, and negative pressure closed drainage can be used as adjuvant treatments to improve the basic condition of the wound and promote wound regeneration and repair (51, 52).

#### 4.4.2 Chronic radiation dermatitis

Manifestations of chronic radiation dermatitis include dermal shrinkage, pigmentation and hypopigmentation, dermal hardiness, dermal ulceration, capillary dilatation, and fibrinisation (53). Due to poor local blood flow, radiation ulcers can easily lead to infection and the wound progressively deepens. Conservative treatment is often ineffective (54). Clinical treatment requires complete debridement to remove ulcer tissue and surrounding fibrotic skin in the first stage, and a musculocutaneous flap with abundant blood flow to fill the wound in the second stage to achieve functional reconstruction and aesthetic appearance (55). Different from other wounds, radiation ulcer has the possibility of recurrence, mostly caused by incomplete debridement. In addition to necrotic tissue and surrounding fibrotic tissue, radioactive injury involving bone, blood vessels and other important tissues should also be resected accordingly, rather than palliative debridement. Of course, this debridement mode will cause large skin soft tissue defects and bone defects. According to the defect site, extent and depth, the corresponding flap and artificial material were selected to reconstruct the bone contour and soft tissue coverage. Histological biopsies may be required for slow refractory skin ulcerations and suspected neoplasms to confirm the diagnosis and rule out the possibility of secondary skin tumours. In addition, fat transplantation can effectively improve skin fibrosis and prevent ulcers (56).

At present, the prevention and treatment of RSI are limited, and there is an urgent need to develop new drugs, such as antioxidants and senescent cell scavengers that scavenge free radicals and reduce oxidative stress damage. In recent years, the field of regenerative medicine has made significant progress, and the application of stem cells and exosomes in RSI therapy has great potential. At the same time, the use of biological materials and regenerative medicine technology to develop multi-functional wound dressings to protect the wound and accelerate healing is also a new method that many scholars are exploring. In addition, the study of low-dose radiation therapy to reduce the occurrence of radioactive skin damage at the source is a key issue that we need to pay attention to.

### 4.5 Strengths and limitations

Using VOSviewer and CiteSpace, this study conducted a systematic bibliometric analysis; evaluated research trends, major research countries, major research institutions, and authors in the RSI field; identified the current research hotspots of RSI; and provided valuable insights for future research directions. However, this study had some limitations. The included studies were only from the WoSCC database and published between 2004 and 2023. This may have uncovered insufficient research literature, affecting the accuracy of the results.

## 5 Conclusion

This study conducted a systematic bibliometric analysis of RSI publications from 2004 to 2023 and identified trends in RSI publications, major research countries, institutions, journals, authors, and keywords, thus revealing the future development directions and research hotspots of this field. This study provides a valuable reference for future RSI research.

## Data Availability

The original contributions presented in the study are included in the article/supplementary material. Further inquiries can be directed to the corresponding author.
